# Hidden Communities of Practice in Social Media Groups: Mixed Methods Study

**DOI:** 10.2196/14355

**Published:** 2020-03-24

**Authors:** Kara Skelton, Retta Evans, Jenna LaChenaye

**Affiliations:** 1 Department of Health, Behavior and Society Johns Hopkins University Baltimore, MD United States; 2 School of Education University of Alabama at Birmingham Birmingham, AL United States

**Keywords:** online social support, breastfeeding, social media, social support system

## Abstract

**Background:**

Although most US mothers initiate breastfeeding, suboptimal breastfeeding rates still exist. Although breastfeeding is a complex process, social support has been linked with increases in positive breastfeeding outcomes. Recent technological advances, including the development of social networking sites, provide mothers with convenient access to a unique array of audiences from which to seek advice about parenting, including breastfeeding. However, little is known about how the use of the sites—specifically groups centered around breastfeeding—influences breastfeeding knowledge, attitudes, or behaviors.

**Objective:**

This mixed methods study aimed to explore utilization of an existing probreastfeeding Facebook group and how utilization influences breastfeeding-related knowledge, attitudes, and behaviors.

**Methods:**

Participants were recruited online through Facebook wall posts from within the existing group. Mothers aged between 18 and 50 years who were pregnant and intended to breastfeed, were currently breastfeeding, or had recently weaned their infant in the past 3 years were eligible to participate. Participants engaged in online focus group discussions (n=21) and individual interviews (n=12). Inductive content analysis of qualitative data led to the conceptualization and contextualization of a breastfeeding community of practice (COP). Using qualitative results, a quantitative survey was then developed to assess the prevalence of qualities of a COP as well as how COP usage influenced breastfeeding-related attitudes and knowledge. A total of 314 mothers completed the online survey.

**Results:**

Qualitative findings showed an overall sense of community, with subthemes of group trust, interaction, and the promotion of breastfeeding. A majority (287/314, 91.5%) of mothers initiated breastfeeding, with 69.0% (216/314) of mothers reporting exclusive breastfeeding their infant at 6 months. Approximately 98.5% (309/314) of mothers reported that the Facebook group captured and stored knowledge; therefore, information could be easily accessed and applied. In addition, 96.2% (302/317) of mothers reported that the Facebook group motivated them to share breastfeeding-related knowledge.

**Conclusions:**

The results suggest that this existing probreastfeeding Facebook group exhibits characteristics of an online COP, which was organically formed. Utilization of the Facebook group, in the context of an online COP, could be beneficial in impacting breastfeeding-related knowledge, attitudes, and behaviors. However, further examination and exploration of breastfeeding COPs, including using this type of model as a method of lactation support or as a telemedicine framework, is a clear need.

## Introduction

### Background

Decades of research have proved breastmilk to not only be the optimal source of nutrition for infants for the first 6 months of life but to also have numerous maternal health benefits. The vast research on the benefits of breastfeeding has lead national child health organizations, including the American Academy of Pediatrics, the World Health Organization, and the Academy for Breastfeeding Medicine, to recommend exclusive breastfeeding for the first 6 months of life, with continued breastfeeding for at least one year and thereafter so long as mutually desired by the mother-infant dyad [1-3]. Despite these recommendations, subpar breastfeeding rates exist in the United States. Although approximately 83% of infants were ever breastfed, only an estimated 24.9% of these infants were exclusively breastfed at 6 months [4]. Disparities exist for breastfeeding rates in the southeast, with breastfeeding initiation rates as low as 63.2% and 6-month breastfeeding exclusivity at an estimated 13.0% for Mississippi. Although Georgia had the highest prevalence in the southeastern states of exclusive breastfeeding at 6 months (22.1%), it is still lower than the national average [4].

The high initiation rate, but low duration of breastfeeding exclusivity rates in the United States, may indicate that mothers lack the necessary support to continue in their breastfeeding journey. Breastfeeding mothers are faced with a plethora of factors that can contribute to high stress in the postpartum period, including lack of sleep, unclear expectations, and the constant learning associated with breastfeeding [5-8]. Just as there are many factors influencing a women’s intention and ability to breastfeed, there are also many ways breastfeeding mothers can be supported through during breastfeeding. Breastfeeding mothers can be actively supported by their partners, families, communities, employers, and peers. Larger-level influences of breastfeeding support include policies, such as paid maternity leave, and insurer-provided lactation support. However, it takes more than active support for breastfeeding mothers to initiate and maintain breastfeeding; maternal self-efficacy, confidence, and anxiety also play a large role. Access to social support, including women-to-women support groups, during the postpartum period has been linked to better maternal health and child health outcomes, including increases in maternal confidence and relationship satisfaction (for both partner-to-partner and parent-child interactions) and decreases in emotional stress [5,9-11]. Furthermore, a recent meta-analysis of social support interventions found these types of interventions to increase breastfeeding initiation by 86% and exclusive breastfeeding by 20% [12].

With recent sociotechnical trends regarding social networking sites (SNSs) and use by mothers, there is a growing field of research centered around the juncture of motherhood and technology. These mechanisms of social interaction in SNSs include peer-to-peer support, knowledge gaining and sharing, establishing friendships, and a sense of belonging, all of which can disappear during the transition to motherhood [11,13,14]. In addition to ease of use, SNSs are convenient and provide mothers with access to a unique array of audiences from which to seek advice about parenting, including infant feeding [11,15,16]. Social media groups, a subset of certain SNSs, rely on user-generated content (UGC) for interaction among users. Existing Facebook groups can be both broadly focused on motherhood and parenting, or more specialized, focusing on one parenting area, such as sleep training or breastfeeding. When social media groups focus on the promotion of one feeding type, such as breastfeeding, it has the potential to create an organically formed community of practice (COP).

Although originally developed as a theory for situational learning, COPs have transformed over the past few decades beyond this meaning [17]. In addition, COPs have expanded beyond previous geographical limitations, presenting an opportunity for the creation of virtual communities based on a shared practice, such as peer-to-peer communities centered on motherhood [18], including Facebook groups. For this study, we define COP as “groups of people who share a concern or passion for something they do and learn how to do it better as they interact regularly” [18]. At their core, COPs are formed by people who engage in a collective learning process and have 3 crucial, defining characteristics: (1) the domain, (2) the community, and (3) the practice [18]. Embedded within COPs are key activities, such as joint problem solving and skill building, which can enhance the formation of social ties [19]. Additional examples of activities include knowledge mapping, requests for information, and advice seeking. When individuals engage in a COP, knowledge flows freely, which supports both knowledge sharing and knowledge seeking behaviors [18,20]. Within the realms of virtual COPs lies an essential component, UGC, which is member generated, and retains components of offline communication [21].

### Objective

The recent growth of UGC embedded within social media groups elicits a need to further understand the social support and communication dynamics in these virtual communities. Although numerous studies have laid the foundation for SNSs, including Facebook groups, as a community building tool, there is a lack of knowledge about how these types of online communities can impact breastfeeding-related outcomes, with multiple studies calling for research on the relationship between social media and breastfeeding [12,15]. Only in recent years have these types of online support mechanisms been explored for the transition to motherhood, with limited research examining how they could provide breastfeeding support [14,22,23]. To address this gap, this research aimed to explore the utilization of an existing probreastfeeding Facebook group within the context of a virtual COP and how utilization may influence breastfeeding-related knowledge, attitudes, and behaviors.

## Methods

### Study Design

This mixed methods study used a sequential exploratory design [24], which consisted of two critical phases: (1) the qualitative phase and (2) the quantitative phase, with the first phase iteratively guiding the second phase of the study. The exploratory design was particularly helpful in achieving the aims of this study, as little information was known about the conceptualization of a phenomenon and how to measure potential key variables. The emphasis in this design was given to the qualitative strand, as it played an essential role in informing the design of the quantitative phase. This study was conducted with the Institutional Review Board approval and oversight (REC300000306).

### Setting and Sample

One existing probreastfeeding Facebook group was used to explore the qualitative and quantitative phases of the study. This group was selected because of the large number of members (>6300), their probreastfeeding approach (as designated by the title of the group), and accessibility to the group (US based). This Facebook group originally stemmed from an in-person support group based at a midsized hospital in Birmingham, Alabama, and was created in 2012. However, there are no restrictions for joining the group; any and all breastfeeding moms are welcome, according to the Facebook group description. As such, the group includes mothers from all over the southeast. There are five administrators of the group, some of whom are International Board Certified Lactation Consultants (IBCLC) and others who do not have any professional training but are experienced in breastfeeding, either from feeding their children or from other experience (eg, work experience as a labor and delivery or neonatal intensive care unit nurse or from being a lactation counselor or dietician).

**Figure 1:**
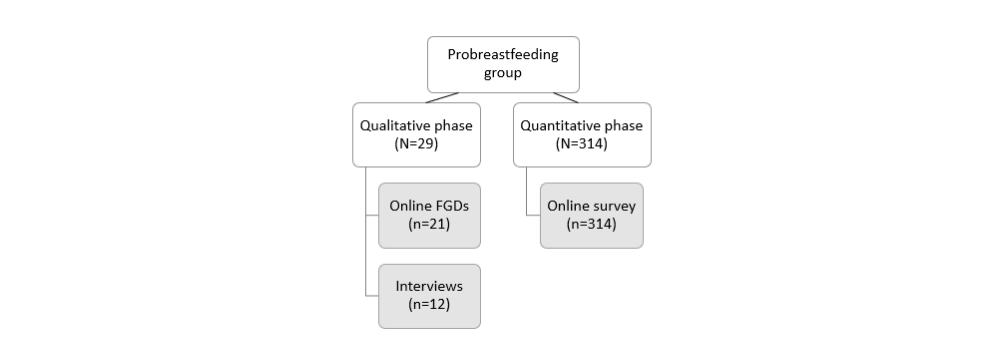
Participant flowchart. FGD: focus group discussion; UGC: user-generated content.

Study participation was limited to women who were existing members of this Facebook group. Mothers between 18 and 50 years of age who were pregnant and intended to breastfeed (mixed or exclusively), were currently breastfeeding (mixed or exclusively), or had recently weaned their infant in the past 3 years were eligible to participate. Mothers were excluded if they had never breastfed a child, were pregnant and intended to only formula feed, or had weaned their infant off breast milk more than 3 years before recruitment. Please see Figure 1 for a participant flowchart that outlines the number of participants for both the qualitative and quantitative strands of the study.

### Study 1: Qualitative Strand

The following research questions guided the qualitative strand of this study: (1) How does the utilization of the probreastfeeding Facebook group support breastfeeding mothers? and (2) Are there COP activities present in the probreastfeeding Facebook group? To fully answer this research question, it was necessary to first broadly explore the overall usage within the social media group through online focus group discussions (FGDs) and further develop emergent themes through individual follow-up interviews. Group posts within the probreastfeeding group were analyzed using USG analysis to examine if and how COP activities were manifested.

#### Qualitative Instrument Development

To structure the online FGDs, we created a focus group guide that consisted of open-ended questions broadly exploring why mothers use social media groups, their perceptions of utilization on breastfeeding-related outcomes (eg, knowledge, education, behaviors, and duration), and perceived barriers to breastfeeding encountered in the virtual realm (see Multimedia Appendix 1).

Individual interview data collection included the creation of a separate guide developed to further explore topics brought up by mothers in the online FGDs. This guide was developed to be comprehensive of the themes derived from the online FGDs but open ended enough to allow interviewees to describe their experiences in sufficient detail. Topics explored in the interview guide included returning to work, positive and negative facets of social media (both in general and specifically for the probreastfeeding social media group), and if participants felt social media group usage had influenced their breastfeeding relationship and why. The following questions were included in the qualitative interview guide: (1) How do you think the probreastfeeding group has impacted your breastfeeding relationship?, (2) What about the other social media groups?, (3) Discuss a time that a social media breastfeeding group has impacted a decision or choice you made with regard to breastfeeding?, (4) What are some barriers or pitfalls to using social media to post or interact with other mothers about breastfeeding?, and (5) How would you describe the information posted in probreastfeeding group with regard to accuracy? For each question, exploratory prompts were used to guide the interview and encourage greater depth of response from participants.

#### Qualitative Recruitment

We used convenience and snowball sampling to recruit mothers via a series of wall posts within the Facebook group in the fall of 2017. The series of posts shared information about the aims of the qualitative portion of the study and asked for their interest in participation. Mothers who responded to the recruitment posts were first asked to participate in one of the three online FGDs. The online FGDs were secret online groups consisting of 6 to 9 mothers, as is the best practice for focus group formation. Once the slots for online FGDs were filled, mothers were asked to participate in interviews. Slots for both focus groups and interviews were filled within 48 hours of the first recruitment post. From the wall post, 37 women were recruited and were eligible to participate. Of the 29 participants who consented for the qualitative strand of this study, 21 participated in the online FGDs, with 12 mothers returning to complete follow-up individual interviews. Although there were 22 participants randomized into online FGDs, 1 participant did not engage or post and was excluded from the analysis. There were 4 mothers who were asked to participate in both the online FGDs and interviews to advance our understanding of topics brought up in the online FGDs. Participants were given a US $10 Amazon gift card for their participation in either the online FGDs or interviews.

#### Qualitative Data Collection

##### Online Focus Group Discussions

After informed consent was obtained, online FGD participants were asked to complete a demographic questionnaire and then randomized into the online FGDs. Online FGDs were facilitated by a trained focus group moderator within the secret online group. An asynchronous approach to the online FGD allowed participants 4 days to read and respond to the initial posts (questions from the online FGD guide) as well as to respond to and interact with other posts in the group. The researcher posted all questions in the secret group ahead of time to enable participants to respond at their convenience. However, the moderator engaged with participants during the online FGDs through the use of prompts to encourage elaboration on responses. Field notes were made during the online FGDs. Posts and responses from each online FGD were copied and pasted into separate documents. Reflection of utilization of the online FGDs for this study showed that this virtual technique was effective in including this sensitive population in qualitative research [25]. Please see Table 1 for online FGD engagement.

**Table 1:** Online focus group discussion engagement characteristics.

Engagement	Online FGD^a^ 1 (n=6), n	Online FGD 2 (n=9), n	Online FGD 3 (n=6), n
Total engagement	151	244	167
Posts	47	78	46
Responses	22	43	42
Likes	82	123	79

^a^FGD: focus group discussion.

##### Interviews

To conduct the interviews, participants provided the interviewer with a time that was convenient for them. Eight of the interviews were conducted in person, with the remaining 4 occurring via Skype. Before the start of the interview, consent was obtained from participants. Interviews were then conducted using the interview guide developed from the synthesis of the online FGD findings. On average, interviews lasted 34 min, but ranged from 17 to 49 min. The interviewer made field notes for each interview. Interviews were audio recorded and then transcribed into separate Word documents.

##### User-Generated Content Analysis

To calculate the sample size for the UGC analysis, the Facebook group was monitored over a 14-day period to get a weekly posting average for a *typical week*. A typical week was considered to be a week in which there were no holidays or school breaks, which could have caused posting to fluctuate. Weekly posting averages were 176 and 188 posts for the first and second weeks, respectively. To obtain a 25% coverage area of average weekly postings, we calculated a random sample size of 44. Any post on the Facebook group page seeking breastfeeding-related advice during a 7-day period was eligible for inclusion, regardless of the number of comments. We collected 44 posts over a 7-day period in November 2017. In addition to the content of posts within the Facebook group, we collected time of posting, number of likes, and number of comments.

#### Synthesis

Online FGDs and interview transcripts were analyzed using inductive qualitative content analysis. NVIVO version 10 qualitative data analysis software (QSR International, Melbourne, Australia) was used for qualitative synthesis. We used in vivo coding initially for each phrase of the transcript as an approach to stay true to the data, as this approach used participants’ own words [26,27]. This methodology is preferred when qualitative data are fragmented, as they are in online FGDs [26]. The coded data were then coded and organized into categories and themes, which led to the creation of a preliminary analysis results document, which was then shared with participants. This member checking was conducted to verify that the researchers’ interpretation of the data was accurate. The identified themes were accepted by all 7 participants who were invited for member checking; no changes were suggested by participants during this process.

### Study 2: Quantitative Analysis

For the quantitative strand of this mixed methods study, we used the following research questions: (1) How does social media group usage impact breastfeeding-related knowledge, attitudes, and behavior? and (2) Can the existing probreastfeeding social media group be considered an online COP?

As qualitative results of study 1 were used for the development of an online questionnaire within the sequential exploratory mixed methods design, the themes and context (eg, community, shared experiences, and trust) revealed in the first phase of this study, in combination with a thorough literature review on each theme, led to the creation of a series of constructs to be included in the online survey. Thus, the quantitative instrument was developed based on the qualitative results. The qualitative analysis revealed a clear need for grounding the online questionnaire in the social capital theories, which was included through the use of social capital scales. In addition, there was a need to include the following content in the questionnaire: social media group usage; social media factors influencing mothers’ breastfeeding-related knowledge, attitudes, and behaviors; and the presence of aspects of a COP within the probreastfeeding Facebook group. After a second literature review to find validated measures of the constructs and content, a survey instrument codebook was created, which mapped the content of the survey, response options, coding schema, source, and validation data.

As there was no published instrument measuring a social media group–based COP, we adapted 3 existing scales: the 2011 survey of Asian Development Bank–Hosted Communities of Practice [28], the Breastfeeding Self-Efficacy Scale-short form [29], and the Iowa Infant Feeding Attitudes Scale [30]. Mothers were also asked about their youngest infant’s breastfeeding outcomes. The survey itself took, on average, less than 15 min and was designed to use language to make participants feel comfortable. No identifiable information or personal health information was collected from participants. Adapted scales were evaluated for psychometric properties and found to have internal consistency (alpha=.72). The full results of this psychometric evaluation, including factor analysis of scales, are forthcoming in a separate manuscript.

#### Recruitment

During the spring of 2019, two recruitment posts within the probreastfeeding Facebook group were used to recruit online survey participants. These posts provided a brief description of topics included in the online questionnaire and included a direct hyperlink to the online questionnaire in Qualtrics. Participants were screened for inclusion through a three-item screener with skip logic embedded within Qualtrics before consent was obtained. Participants who completed the entire online questionnaire were automatically entered to win 1 of the 2 US $50 Amazon gift cards.

#### Data Collection

Once participants consented, they were able to move forward onto the online questionnaire. A total of 314 mothers completed the online survey.

#### Data Analysis

All surveys were completed via Qualtrics, an online research and experience software. Qualtrics securely hosted all survey responses until downloaded into a .csv file. All data files were stored on a password-protected computer. Preliminary validation testing was conducted for developed COP scales to determine internal consistency via exploratory factor analysis. The results of the exploratory factor analysis found the developed scales to have internal consistency. For this study, we will report only basic descriptive statistics, including proportions, frequencies, means, and standard deviations for the online survey. We also report mean scale scores and associated standard deviations for the developed COP scales. All descriptive analyses were conducted using SPSS Statistics version 22 software (IBM, Armonk, New York) [31].

## Results

### Study 1

#### Demographics

Of 29 mothers who participated in the qualitative strand, 2 were currently pregnant, 25 (86%) were currently nursing, and 4 (14%) had weaned their infant in the past 12 months. A majority of mothers were white (25/29, 86%) and worked either full time or part time (25/29, 86%). The mean age of participants was 29.7 years, with a range of 23 to 40 years. Moreover, 41% (12/29) of the participants had a high school diploma and some college degree, with 58% (17/29) of the participants reporting attainment of at least a bachelor’s degree. The majority (21/29, 75%) of mothers in the qualitative strand had been in the probreastfeeding Facebook group for 6 months or longer.

#### Overarching Theme: Creating Community

The results from FGDs and interviews revealed an overarching theme of community across participants’ reported experiences within the Facebook support group. Overall, mothers felt like the probreastfeeding Facebook group was a place where they were able to bond with others by uniting strangers together around one topic: breastfeeding. Participants described their relationship with the group as being “always nice to have a place to go where you are ‘understood’.” They also reported appreciating the group’s ability to “normalize not only breastfeeding but also the troubles that surround breastfeeding moms. It brings us together!”

#### Shared Experience in Breastfeeding

One subtheme derived from the qualitative analysis was the shared experience among members in the social media group. As 1 participant stated, “Posting [on the social media group] allowed me to reach other moms that were in similar situations or had similar issues.” Many mothers mentioned the immense support they felt within the group and their appreciation for this support, which they may have otherwise not received. Furthermore, many mothers mentioned that they did not have anyone within their immediate social network who had breastfed, which left them with a desire to find others who had breastfed. One mother shared:

With no mothers in my own family who breastfed, the number of women to whom I can ask questions is very limited. Social media broadens that pool.

Another mother described the importance of social support during breastfeeding and how this social media group provided that support for her, stating:

I believe it is important to interact with other mothers dealing with the same issues and concerns. It’s important for a breastfeeding relationship to have some sort of support and social media can provide that to an extent.

Mothers described their experiences within the Facebook group as mostly uplifting and positive. One mother described her experience with the group and how it helped her to not give up on breastfeeding:

I knew I had someone to ask questions to, so it allowed me to not give up when I struggled. I felt like lots of other moms had some of the same struggles I did. I liked that in a world that is only slowly accepting open breastfeeding or accommodations, it felt so normal and so celebrated in this group.

Another mother described similar encouragement received from the group, stating that:

The support I received from a social media group was invaluable. I was encouraged to never quit on a bad day.

#### Social Interaction

Most mothers reported that they were interacting within the group in some fashion (23/29, 80%), primarily in the form of knowledge sharing (20/29, 70%) and asking questions (19/29, 66%). For those who did not report regular interaction, the reasons that were cited were because they “searched within the social media group to see if the question had already been answered” or had “recently weaned their infant and no longer breastfed.”

Mothers reported that they enjoyed sharing information within the group, feeling that “it’s my job, as a member of the group, to comment with a carefully-worded response that is uplifting and kind.” In addition to sharing information within the social media group, mothers enjoyed having real-time responses to inquiry and associated feedback:

If I have a question about breastfeeding, I have hundreds of women who have experience breastfeeding at my fingertips. I have direct access to at least one IBCLC and several experts. Other moms with the same question can read the post and benefit from the information.

#### Confidentiality and Trust

Participants also found a strong sense of confidentiality within the group. Mothers reported that they felt fellow members were focused on promoting best practices for breastfeeding and provided encouragement and support, which led them to developing a strong sense of trust and nonjudgment within a group composed predominantly of strangers. One aspect of trust embedded within this community was the quality of breastfeeding information throughout the group. Mothers felt like the information received in the group was consistent with evidence-based practices for breastfeeding, especially in comparison with other parenting and mom groups they were a part of:

In the group, the postings almost always adhere to AAP guidelines and the admins even provide evidence-based articles and studies to support the guidelines. In other groups, I feel most of the advice is very ill-advised in all respects, both in regard to AAP guidelines and in regard to other general breastfeeding and pumping advice.

Many mothers felt this probreastfeeding Facebook group was of high quality, often comparing it with others they considered less trustworthy. One mother shared her broader experience with social media groups, including why she chose to leave other groups:

The experience with social media regarding breastfeeding strongly depends on your social network and which group(s) you’re a member of. There was one group that I had initially joined, but later left due to the fact that I felt it put more of a negative stigma on breastfeeding, rather than normalizing it and bringing positivity to it, despite the fact that it was intended as a “pro-breastfeeding” site. Others I’ve left due to gross misinformation and terrible advice.

A large portion of the discussion for both online FGDs and interviews centered around the trust and confidentiality within the Facebook group, indicating this type of group as a rich place for knowledge sharing.

#### Activities of a Community of Practice

The USG content analysis revealed that within the probreastfeeding Facebook group, not only were key themes brought up about community, but key COP activities were also present. These COP activities included reciprocity, joint problem solving, and skill building, to name a few. Textbox 1 shows descriptions and specific examples of COP mechanisms embedded within this probreastfeeding group.

**Community of practice activities present in the probreastfeeding Facebook group.**

Problem solving

- “Any tips for a very sore and cracking nipple situation? It hurts so bad to latch”

Requests for information

- “When baby is on solid foods 3x a day, how much breast milk should he be getting?”

Seeking experience

- “Just had my baby at midnight via emergency c section. He is in the NICU. Already pumping. Any advice to make sure I do the best for my supply until I can start feeding him?”

Reusing assets

- “We love the MommyMeds app from the Infant Risk Center- download it!”

Coordination and synergy

- “We will be teaming up with Babywearing International of for a baby wearing meet-up!!!”

Mapping knowledge and identifying gaps

- “I know occasionally we have mamas post they are stranded without their pump or certain parts. What if we had ‘pumping stations’ around town?”

### Study 2

Sociodemographic characteristics of mothers who completed the online survey, including maternal characteristics, are shown in Table 2. Approximately 91.5% (287/314) of mothers reported breastfeeding initiation, with 69.8% (216/314) of mothers reporting exclusively breastfeeding their infant at 6 months. Less than half (112/314, 35.6%) of the mothers reported taking a breastfeeding prenatal class. Almost half (140/314, 44.8%) of the mothers had been in the Facebook group more than 12 months, with 18.1% (57/314) and 37.1% (116/314) being in the group between 6 to 12 months and less than 6 months, respectively.

**Table 2:** Demographic characteristics of the participants who completed the online questionnaire (N=314).

Characteristics		Values
Age (years), mean (range)		29.85 (19-42)
**Race, n (%)**		
	African American	4 (1.3)
	American Indian or Alaskan Native	2 (0.6)
	White	300 (95.5)
	Other	8 (2.5)
**Marital status, n (%)**		
	Single, never married	7 (2.2)
	Married	284 (90.4)
	In a monogamous relationship	23 (7.3)
**Education, n (%)**		
	High school diploma or some college	106 (33.8)
	Bachelor’s degree (4 years)	113 (35.9)
	Master’s degree	65 (20.7)
	Professional degree (Juris Doctor and Doctor of Medicine)	30 (9.5)
**Working status, n (%)**		
	Full time or part time	234 (74.5)
	Not working	80 (25.4)
Previously breastfed a child, n (%)		129 (41.1)
Full-term infant, n (%)		229 (77.6)
**Interaction with social media group, n (%)**		
	Ask questions	240 (76.4)
	Give advice	225 (71.6)
	Does not interact regularly	51 (16.2)

The COP scale asked participants to answer how they agreed with statements about the probreastfeeding social media group. Responses were in a Likert scale format, ranging from very strongly disagree (1) to strongly agree (4). Please see Table 3 for all items included in the scale to asses social capital within the probreastfeeding group and associated means and standard deviations.

A majority (257/262, 98.1%) of the mothers agreed or strongly agreed that “there was a clear focus on breastfeeding” within this social media group. When asked if mothers felt the Facebook group “built knowledge sharing and learning into the group,” approximately 99.2% (260/262) of mothers reported that they either agreed or strongly agreed with this statement. Approximately 98.4% (258/262) of mothers reported that they either agreed or strongly agreed that the social media group captured and stored knowledge; therefore, it could be easily accessed and applied. Furthermore, 96.6% (253/262) of mothers reported that the social media group motivated them to share breastfeeding-related knowledge. There were 97.0% (254/262) of women who reported that they felt the information and advice shared within the group is accurate. Approximately 96.2% (252/262) of participants felt the group helped them to achieve their breastfeeding outcomes. Only 85.5% (224/262) of mothers reported that they agreed or strongly agreed with the statement, “this Facebook group helps me to build my relationship with others.”

**Table 3:** Scale to assess social capital within the probreastfeeding group descriptive statistics.

Item	Value, n (%)^a^	Value, mean (SD)
Has a user-friendly communication platform	260 (99.2)	3.32 (0.48)
Build knowledge sharing and learning into the group	260 (99.2)	3.30 (0.47)
Capture and store knowledge so it can be easily applied	258 (98.4)	3.32 (0.50)
Helps me achieve better breastfeeding outcomes	258 (98.4)	3.27 (0.49)
Benefits my breastfeeding outcomes	257 (98.1)	3.27 (0.50)
Helps to build my confidence	257 (98.1)	N/A^b^
Represents a common area of interest for many mothers in the group	257 (98.1)	3.15 (0.40)
There is a clear focus on breastfeeding	257 (98.1)	3.13 (0.37)
Benefits my breastfeeding relationship	257 (98.1)	3.23 (0.48)
Is driven by the willingness of members to participate	255 (97.3)	3.28 (0.53)
I trust the group members	254 (97.0)	3.38 (0.56)
The information/advise shared is accurate	254 (97.0)	3.37 (0.56)
Motivates me to share breastfeeding-related knowledge	253 (96.6)	3.18 (0.46)
The group helped me to achieve my goals for breastfeeding	252 (96.2)	3.26 (0.53)
Gives me a sense of empowerment	252 (96.2)	3.25 (0.53)
Gives me a sense of belonging	250 (95.4)	3.25 (0.56)
Breaks down communication barriers among members	247 (94.3)	3.34 (0.61)
Helps me to build relationships with others	224 (85.5)	3.25 (0.71)

^a^n is reported as the number of women who agreed or strongly agreed with the statement.

^b^N/A: not applicable.

## Discussion

### Principal Findings

The combined approach to this study enabled us to fully explore an existing probreastfeeding Facebook group as an organically formed online COP and to elaborate both qualitatively and quantitatively on how these mothers felt their involvement with the social media group effected their breastfeeding journey. Looking at both the qualitative and quantitative findings of the study, the following key findings were made: (1) breastfeeding mothers reported the peer-to-peer support from the probreastfeeding Facebook group to be invaluable and that the group itself is a resource for knowledge and interaction that consequently impacts the breastfeeding relationship and (2) this specific Facebook group organically formed a COP, as demonstrated through the presence of key characteristics within the group. These conclusions regarding the organic formation of this online community as a COP would not have been possible without the interaction of the two strands (qualitative and quantitative) of this study, which is a strength of this study. This study helps to fill gaps in the current field regarding social media group usage and breastfeeding practices.

To elaborate on the notion of a hidden COP, we found the following key tenants of a COP in the probreastfeeding Facebook group: (1) commitment, that is, a shared domain (the social media group); (2) a virtual community that distinguishes its members from others (mothers); and (3) the practice (breastfeeding) [18]. Through engagement in joint activities, stories and experience, and knowledge sharing, members of the COP were able to support one another in their breastfeeding journey. Also consistent with communication within a COP, breastfeeding knowledge flowed freely in this Facebook group, without social norms of reciprocity. A shared repertoire of resources is also essential for sharing the practice, which was available to the COP through current and past posts and responses within the Facebook group. The shared domain, practice, and community were dynamically integrated into the probreastfeeding Facebook group; therefore, we can say it is indeed a hidden COP. Within the context of this breastfeeding COP, mothers reported that their breastfeeding-related questions or concerns were addressed with information consistent with clinical breastfeeding guidelines and national recommendations for breastfeeding.

Participants mostly reported positive feedback around the probreastfeeding social media group. More importantly, mothers also reported high agreement with statements showing the breastfeeding group not only as a COP but also as a mechanism of support, empowerment, and trust for all things breastfeeding. Examining breastfeeding prevalence rates in the probreastfeeding Facebook group compared with the national average, there was a higher prevalence of breastfeeding exclusivity at 6 months among mothers in the Facebook group (69%) compared with national data from the 2018 Breastfeeding Report Card (24.9%) [4]. Furthermore, 91.5% (287/314) of mothers who completed the online survey reported initiation of breastfeeding, which is also higher than the national average of 83%. The rates of breastfeeding initiation and exclusivity among members of this probreastfeeding group may indicate that group membership, and specifically the COP activities identified within the Facebook group, influences breastfeeding duration. However, further statistical analyses are warranted to examine differences in prevalence rates.

Findings of this study may have been influenced by the presence of group moderators who were certified lactation consultants, as they deleted posts citing outdated or misinformation. This is a powerful characteristic of the Facebook group under study because although knowledge sharing within social media groups is intended to help, it can often lead to confusing mothers when not aligned with clinical guidelines. There are multiple ways results from this study can impact clinical practice and implementation of breastfeeding-related programming. As mother-to-mother support groups are well known to provide opportunities for breastfeeding mothers to support breastfeeding through sharing of experiences, discussion in overcoming challenges, and through a sense of belonging [10,32-34], these types of groups could be the foundation for evolving telemedicine and electronic health models for lactation support, especially ones that are moderated by professional lactation clinicians, but also include mothers. This type of telemedicine model could be a way for hospitals and lactation consultation practices to expand their reach to mothers, ensuring access to reliable information online. This type of virtual COP could also be a place for referral to other health care professionals when there are other issues suspected (eg, referral to a pediatric dentist if posted picture of baby’s frenulum showed a tongue tie and referral to a psychologist for postpartum depression symptoms). However, more foundational work, including interventions with other breastfeeding-related, social media–related technologies and platforms, should be conducted before the provision of concrete recommendations in this area.

These novel findings also have implications for breastfeeding promotion and practice. Regarding breastfeeding promotion, it seems social media groups, especially those that focus on breastfeeding, can disseminate and promote best practices within the group. This is consistent with a recent study conducted in Australia on the social media group use by a national breastfeeding organization, in which they found that the social media group promoted best practices for its members [14]. Furthermore, this social media group was able to provide critical social support that mothers reported they were not receiving elsewhere. Many mothers, especially those with multiple children, do not have the time to go to in-person support groups because of parental or work-related constraints [25]. However, mothers find interacting with others online to be convenient and can lead to the development of relationships over time. Reaching mothers through virtual communities, especially social media groups, has an immense potential to increase the reach of breastfeeding education and programming. Future research should explore how health care professionals can leverage existing social media, mobile health apps, and emerging technologies to promote breastfeeding and provide mothers with support.

### Limitations

Although there are many strengths to this study, there are also limitations that must be considered. As this was an exploratory study with a small qualitative sample from a group of mothers located mainly in the southeast, results are not generalizable to all breastfeeding mothers who use social media or all breastfeeding groups. This is traditional of qualitative studies, as they are meant to describe and understand the phenomenon of interest. The smaller sample size was also intentional, as qualitative studies are small because of their in-depth nature. As there was only one coder for the qualitative data, interrater agreement could not be tested. However, member checking was performed to validate thematic analysis. In addition, as focus groups rely on the individuals’ perceptions and experiences of social media group use and breastfeeding, these perceptions are also based on sample selection. With regard to sample selection, there is also the potential for self-selection bias in those mothers who participated, meaning those mothers who agreed to participate in the study may have been more likely to see themselves as ideal participants (eg, active participation in the group, success with breastfeeding, and previously breastfed an infant). However, we did have varying degrees of interaction within the group as well as variability in breastfeeding outcomes (eg, exclusively breastfeeding, mixed feeding, and breastfeeding barriers). It is important to note that because of the cross-sectional nature of this study, causality could not be determined.

### Conclusions

This mixed methods study explored a novel area: using existing specialized infant feeding Facebook groups as hidden COPs. Mothers reported that they felt their interaction within the probreastfeeding Facebook group benefited their breastfeeding relationship through the formation of a breastfeeding community that empowered them in their breastfeeding journey. However, the findings presented here are preliminary and descriptive. The examination of this probreastfeeding social media group would not have been possible without the *mixing* of the qualitative and quantitative data. We recommend future studies employ this approach to move the field forward. Further analyses on the data from this study are needed to better understand and determine how social media groups may influence breastfeeding-related outcomes. This also includes research that aims to determine how the formation and utilization of a virtual breastfeeding COP can be replicated in other social media groups or virtual communities as well as to explore the casual relationships between group usage and breastfeeding-related knowledge, attitudes, and outcomes.

## Appendix

Multimedia Appendix 1 Focus group guide.

## Abbreviations

COP: community of practice
FGD: focus group discussion
IBCLC: International Board Certified Lactation Consultant
SNS: social networking site
UGC: user-generated content
