# Supplementary material for: Hidden Communities of Practice in Social Media Groups: Mixed Methods Study
Source: JMIR Pediatr Parent. 2020 Mar 24;3(1):e14355. doi: 10.2196/14355 (PMC7139422; doi:10.2196/14355)
Supplement: Multimedia Appendix 1 [file pediatrics_v3i1e14355_app1.docx]

**Multimedia Appendix 1. Focus Group Guide**

| 1. Have you found that your interaction(s) with social media groups impacts your breastfeeding related intentions, attitudes, outcomes or behaviors? If so, describe how. |
| --- |
| 1. Elaborate on barriers for social media use while breastfeeding and breastfeeding barriers in general. |
| 1. Why, and when do you post in social media groups about your breastfeeding experience? |
| 1. How do you think the pro-breastfeeding social media group impact your breastfeeding relationship? |
| 1. How do you think posting on social media groups will benefit you or your infant while breastfeeding? |
| 1. Are you aware of the AAP evidence-based best practice guidelines for breastfeeding? |
| - 1. How does the content of social media postings, particularly in the pro-breastfeeding Facebook group adhere to the AAP guidelines for evidence-based practices? |
|  |
